# Navigating guidelines and realities: informed free choice in infant feeding for people living with HIV

**DOI:** 10.3389/frph.2026.1817056

**Published:** 2026-04-22

**Authors:** Laura Cox, Ciarra Covin, Monica Hahn

**Affiliations:** 1 Dept. of Family Health Care Nursing, School of Nursing, University of California, San Francisco, CA, United States; 2 The Well Project, Philadelphia, PA, United States; 3 HIVE Clinic and Department of Family & Community Medicine, University of California, San Francisco, CA, United States

**Keywords:** breastfeeding, chestfeeding, HIV, informed free choice, reproductive justice

## Abstract

Recent shifts in U.S. and global HIV perinatal guidelines have reopened long-constrained conversations about infant feeding for people living with HIV (PLWH), including the option of breast/chestfeeding under conditions of viral suppression. While these changes represent meaningful progress, they unfold within a historical and contemporary landscape shaped by medical racism, gender-based oppression, criminalization, and persistent HIV stigma - forces that continue to constrain autonomy and trust in care. In this Perspective, we propose the Informed Free Choice model, a reproductive justice informed framework that moves beyond conventional shared decision-making to explicitly address structural inequities, power dynamics, and the lived realities of families affected by HIV. Drawing on interdisciplinary clinical practice, community advocacy, and participatory training and organizing with women living with HIV, we situate infant feeding decisions within multilevel systems of care and governance. We argue that Informed Free Choice requires not only accurate risk–benefit counseling, but also meaningful involvement of people living with HIV (MIPA), interdisciplinary care teams, trauma-informed and culturally responsive practices, and policy alignment that protects families from coercion, surveillance, and punishment. We conclude by outlining implications for clinical practice, guideline development, and future research, positioning Informed Free Choice as a necessary evolution in perinatal HIV care that aligns scientific advances with reproductive justice and health equity.

## 1 Introduction

Infant feeding decisions among people living with HIV sit at the intersection of biomedical evidence, public health policy, family life, and deeply rooted histories of racialized and gendered control over reproduction. For decades, U.S. clinical guidance framed HIV as an absolute contraindication to breast/chestfeeding, a position that, while grounded in early scientific uncertainty, was operationalized through paternalistic counseling, coercive practices, and, in some cases, criminalization and family surveillance. Importantly, and in contrast to this recommendation, global guidelines have been developed that differ significantly from those of the US, primarily due to limited access to resources such as clean water and formula (1, 2). These approaches disproportionately harmed Black, Indigenous, and other people of color, gender-diverse people, immigrants, and those living in poverty, compounding existing inequities in maternal and infant health and eroding trust in healthcare systems.

We write as an interdisciplinary group of clinicians, researchers, advocates, and people living with HIV who have worked collectively in clinical care, community organizing, training, research and policy engagement to improve perinatal HIV care. As one of our co-authors, a Black woman living with HIV who has navigated pregnancy, delivery, and breastfeeding while virally suppressed, has reflected at national discussions: “I'm not looking for permission. I'm looking for support.” This lived experience grounds our insistence that choice must be both informed and free to be meaningful. Our authorship includes a woman living with HIV who has navigated pregnancy and breastfeeding and serves as an advisor to national protocols, a nationally recognized perinatal HIV physician leader, and an experienced certified breastfeeding specialist (CBS), doctoral candidate, and OBGYN nurse whose scholarly research focuses on infant feeding equity. Our perspective is shaped by years of collaboration within a multidisciplinary working group dedicated to developing guidance for clinicians and families navigating infant feeding options in the context of HIV. This positionality informs both our critique of existing models and our proposal for a reimagined approach.

Through a reproductive justice lens, we propose the Informed Free Choice model as a framework for supporting infant feeding decisions among people living with HIV (PLWH). This model explicitly acknowledges the social, cultural, structural, and intersectional forces that shape decision-making. In doing so, it seeks to transform infant feeding counseling into a rights-affirming, equity-oriented process that centers patient agency and community knowledge.

## 2 Disparities, criminalization, and the legacy of harm

The landscape of the HIV epidemic in the United States (US) continues to disproportionately affect birthing individuals from Black, Indigenous, and People of Color (BIPOC) communities (3). Structural racism, poverty, unstable housing, educational inequities, criminalization, medical mistrust, and systemic oppression amplify disparities in viral suppression, poor maternal and fetal outcomes (4–8).

Since the late 1980s, laws and policies have criminalized PLWHIV, requiring surveillance, rather than individuals entitled to bodily autonomy and family life. As of December 2023, 34 US states have laws that criminalize or control behaviors perceived to pose HIV transmission risk (9). These regulations extend to breast/chestfeeding, with documented cases of Child Protective Services (CPS) involvement, often at the request of the provider (10). Moreover, such interventions disproportionately target BIPOC families, instigating stigmatization and resulting in impacts to mental health, barriers to viral suppression, and limiting the ability to make informed choices (11–14). Stigma and discrimination, often fueled by criminalization, continue to shape how people living with HIV engage in care and make decisions surrounding their birth and infant feeding options.

A historical review of public health policies related to infant feeding and perinatal treatment guidelines reveals a narrative shaped by stigma and bias across multiple axes of power, including race, class, gender identity and sexual orientation. In the early years of the HIV epidemic, public health institutions and healthcare providers largely adopted practices including reproductive coercion, paternalism, criminalization, and a generally punitive approach toward patients labeled as “noncompliant” with providers' directives (15–20). Moreover, organizational guidelines further advanced criminalization and stigma among those living with HIV, including recommendations that discourage pregnancy, which have lasting effects on the HIV community (21). Although the scientific landscape has changed dramatically, the residual effects of these approaches continue to shape patient–provider interactions and institutional practices today.

### 2.1 Evolving guidelines and persistent gaps

Recently, there has been a nationwide paradigm shift regarding infant feeding choices and HIV. In 2023, the Department of Health and Human Services updated its perinatal HIV guidance to recommend a shared decision-making approach that includes the benefits of lactation for individuals with sustained viral suppression and discussion of a less than 1% HIV transmission risk (22). The benefits of human milk for both lactating individuals and infants are well-established in the literature (23–25) and for those living with HIV breast/chestfeeding continues to be recommended by both AAP and the WHO (26, 27), as well as supported by professional organizations including the Academy of Breastfeeding Medicine, the Global Breastfeeding Collective and the Association of Nurses in AIDS Care (28–30).

Despite this progress, implementation gaps and barriers to breast/chestfeeding persist. Recent literature from the US has found a lack of infant feeding policies, withholding of all infant feeding options, and pressure to formula feed from providers (31, 32). Providers report uncertainty, lack of institutional policies, limited interdisciplinary support, and fear of legal or professional repercussions (33). A 2024 survey of providers conducted by the Well Project found that the majority surveyed support the change in infant feeding guidelines and encourage a discussion with people living with HIV about infant feeding options. However, a lack of interdisciplinary collaboration remains a barrier to engaging in a shared decision-making model (34). Moreover, providers surveyed reported institutional barriers to supporting breast/chestfeeding for patients living with HIV, including a lack of explicit policies from institutions (33, 34).

Misalignment among professional organizations that provide guidance and viewpoints are not aligned to include human milk feeding for those living with HIV, further confusion and inconsistency in care. ACOG continues to list HIV as a contraindication to human milk feeding (35) and it took over a year for the AAP to update its guidelines with language similar to that of DHHS, leaving parents living with HIV and providers alike without clear guidance on how to engage in decision-making related to infant feeding (36). These findings underscore gaps in shared decision-making, the need for evidence-based policies, and healthcare provider training that considers unequal power relations, structural constraints, and the historical context that shapes how information is received (10).

### 2.2 Reproductive justice, MIPA, and perinatal HIV care

Reproductive Justice as defined by SisterSong, affirms the human right to bodily autonomy, to have or not have children, and to parent children in safe and sustainable communities (37). This framework serves as a critical lens to the multilevel conditions that enable or constrain meaningful choice, while requiring centering the voices and leadership of women and birthing people living with HIV across the life course.

Meaningful Involvement of People with HIV/AIDS (MIPA) movement has been foundational to the global HIV response, driving advances in policy, research ethics, and service delivery. In the context of infant feeding, MIPA calls for genuine partnership in guideline development, research design, clinical training, and evaluation (38). Evidence demonstrates that when people living with HIV are excluded from these processes, policies fail to reflect lived realities and continue to perpetuate inequities (39, 40).

Applying the reproductive justice and MIPA frameworks to perinatal HIV care, includes how providers counsel patients living with HIV through perinatal care decisions, such as infant feeding, while considering ethical dilemmas, provider bias, criminalization, racism, stigma, and repercussions of discriminatory policies to achieve social justice and foster retention in HIV care (41–43).

### 2.3 Limits of shared decision-making

Shared decision-making, as defined by Elwyn and colleagues, is a process in which patients and providers make a decision together using the best available evidence (44). Fundamentally, shared decision-making aims to center the patient while providing evidence-based information to collectively reach a healthcare decision (45). However, through a reproductive justice lens, social and structural blind spots generate barriers that limit true engagement with shared decision-making.

While shared decision-making was a model developed to place the provider and patient at the center of the decision-making process, several shortcomings of this model are elucidated at the systemic, provider, and patient levels. Systematically, recent evidence highlights gaps in clinical policy and provider education, often leaving the implementation of shared decision making fractured and ineffective (46, 47). Provider-level barriers include misinterpretation of patients' preferences (48, 49), a lack of provider training (46), and a lack of time (50). From the patient perspective, include an unacknowledged priority of patients' needs (51) and a lack of feeling seen and understood (52), and among marginalized communities, a lack of feeling qualified to make certain decisions about their health (53). Empirical studies of infant feeding counseling among PLWH illustrate these dynamics. Many patients report that breastfeeding was never discussed as an option (54), or that discussions were framed in ways that discouraged or implicitly prohibited it (55). Even when providers intend to engage collaboratively, the absence of structural supports and clear institutional backing can result in counseling that falls short of true choice (31, 56).

### 2.4 Beyond shared decision making: the informed free choice framework model

To address these gaps, we propose the Informed Free Choice model (Table 1), grounded in reproductive justice, anti-oppression praxis, and MIPA. Informed Free Choice builds on and substantively extends the shared decision-making paradigm to address inequities that shape infant feeding options for people living with HIV. This model explicitly integrates social and structural drivers of health, community accountability, and patient-defined priorities.

**Table 1:** Shared decision-making vs. informed free choice in perinatal HIV infant feeding.

Dimension	Shared decision-making	Informed free choice
Conceptual foundation	Patient-centered care and evidence exchange	Reproductive justice, MIPA, anti-oppression, patient-centered care
View of autonomy	Individual preference within a clinical encounter	Relational autonomy shaped by social, structural, and historical forces
Role of clinician	Information provider and decision partner	Facilitator, advocate, and protector of patient agency
Attention to power and history	Often implicit or absent	Explicit acknowledgment of racism, criminalization, stigma, and reproductive coercion
Scope of responsibility	Primarily interpersonal (patient–provider dyad)	Multilevel: interpersonal, community, and structural
Community involvement	Limited or consultative	Meaningful involvement and leadership of people living with HIV
Goal	Reach a mutually agreed-upon decision	Enable a decision that is informed, freely made, and safely supported over time

**Figure 1:**
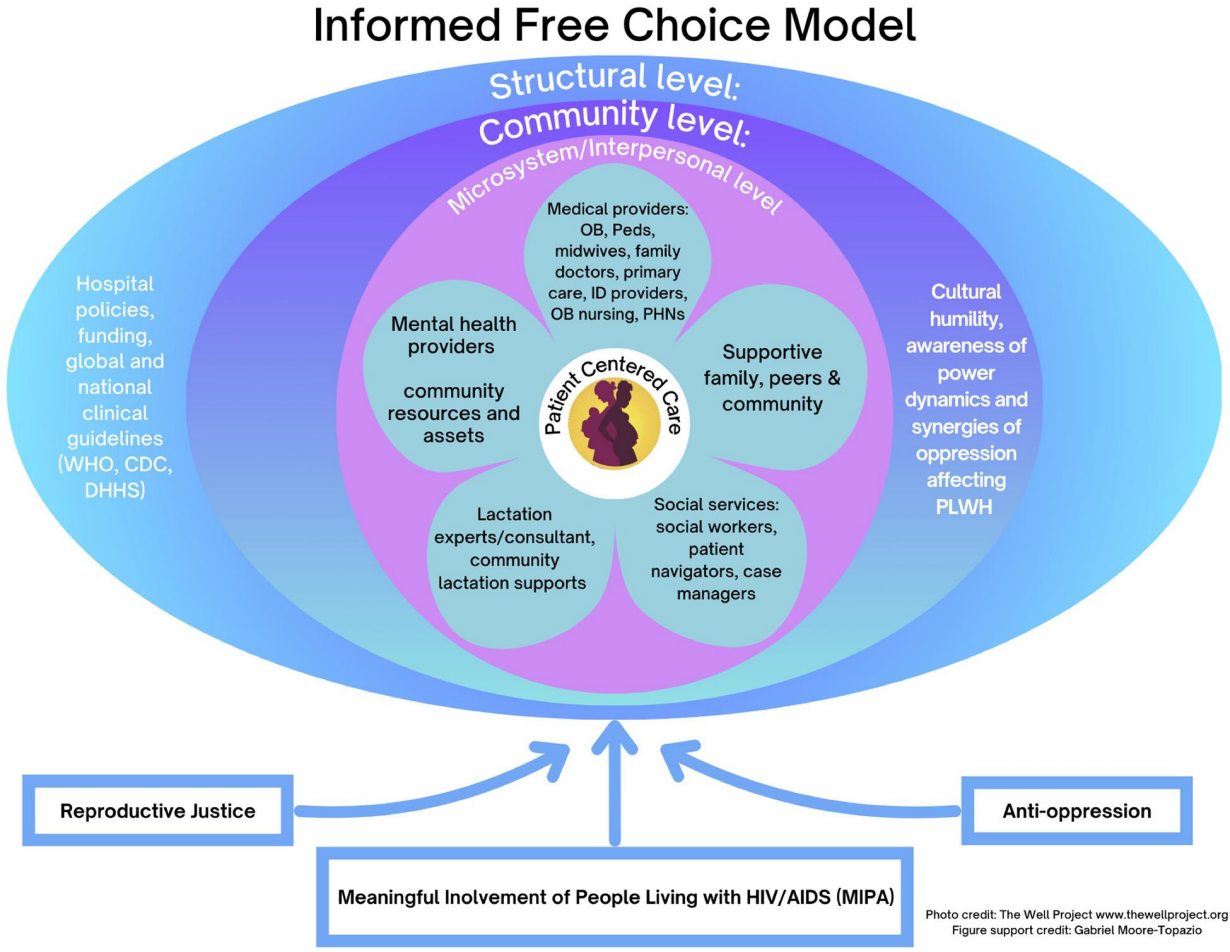
Informed free choice model with patient centered care at the core, layered by interpersonal, community, and structural influences. Arrows highlight reproductive justice, anti-oppression, Meaningful Involvement of People Living with HIV/AIDS (MIPA).

At the center of the model (Figure 1) is the patient, recognized as an expert in their own life. Surrounding this core interdisciplinary microsystem of care that includes perinatal and HIV clinicians, pediatric providers, nurses, lactation consultants, mental health professionals, social workers, case managers, and community-based advocates. This team-based approach facilitates consistent messaging, reduces provider isolation, and supports patients across clinical and community settings. Current findings suggest that including community health care workers in a multidisciplinary team has reduced patient barriers and improved access to HIV care, especially when they work alongside providers (57, 58). Furthermore, improved communication across siloes between physicians, nurse practitioners, and nurses improves provider satisfaction and decreases feelings of burnout (59, 60). Therefore, implementing a multidisciplinary approach in the informed free choice model improves patient outcomes and increases provider satisfaction.

At the community level, Informed Free Choice emphasizes trauma-informed, culturally responsive care, critical awareness of power dynamics, and intersectionality-informed practice. Providers are called to reflect on how racism, sexism, HIV stigma, and criminalization intersect in clinical encounters, and to actively work to mitigate their effects. A trauma-informed approach to patient care and adequate training of healthcare providers can improve patient-centered care and reduce the effects of trauma (61). Training and institutional support are essential to operationalize these commitments.

The macro level includes structural and systemic factors that includes hospital and clinical policies, resource allocation, funding priorities, and professional guidelines in shaping what choices are truly available. Global health research suggests that unclear infant feeding guidelines and mixed messaging from providers and health institutions create barriers to infant feeding decisions among those living with HIV (55, 62). Moreover, at the structural level, factors such as economic policies that underfund lactation initiatives and allow corporate influence that impact lactation rates (63). Alignment across organizations, explicit protections against punitive responses to infant feeding decisions, and investment in lactation and community support services are necessary conditions for Informed Free Choice.

## 3 Discussion

The rapid evolution of HIV science has created new possibilities for infant feeding among PLWH, but realizing these possibilities requires more than updated guidelines. It requires reckoning with the historical harms embedded in perinatal HIV care and committing to models that foreground justice, autonomy, and community leadership. The Informed Free Choice model offers a pathway for aligning clinical practice with reproductive justice by reframing infant feeding counseling as a multilevel, relational, and rights-affirming process. From our collective experience in interdisciplinary care and community collaboration, we have seen how coordinated, respectful approaches can support families in making decisions that align with their values while maintaining engagement in HIV care.

Future research should evaluate the implementation of Informed Free Choice–oriented interventions, including their impact on patient experience, provider satisfaction, infant feeding outcomes, and postpartum retention in care. Participatory and community-engaged methodologies will be essential to ensure that this work remains accountable to those most affected.

### 3.1 Practice implications for clinicians, institutions, and systems

To translate Informed Free Choice into routine perinatal HIV care, we propose three immediate, actionable implications:

- **Embed MIPA in clinical and policy processes:** Institutions and guideline bodies should formally include women and birthing people living with HIV as compensated partners in the development, implementation, and evaluation of infant feeding policies, clinical tools, and training programs. This includes truly community-driven interventions such as community advisory boards which can inform the equitable provision of care.
- **Operationalize patient-centered, rights-affirming counseling:** Clinicians should provide clear, evidence-based counseling on all infant feeding options while explicitly addressing stigma, legal concerns, and historical harms. Counseling should affirm patient expertise, document preferences without coercion, and include plans for longitudinal support that extends well into post-partum care and ensures continuity into primary care, regardless of feeding choice.
- **Align systems to protect and support choice:** Health systems must establish clear policies that support breast/chestfeeding for virally suppressed parents living with HIV, prohibit punitive responses (e.g., unwarranted CPS involvement), and ensure access to interdisciplinary supports such as lactation consultation, mental health care, and community-based advocacy. Supporting interdisciplinary communication is key to unified messaging and continuity of care. Post-partum and infant care should be streamlined and ideally co-located to minimize fragmentation of care, and may strengthen support for families.

One of the earliest and most fundamental decisions a parent makes is how to feed their child. For those living with HIV, the right to decide how to feed their infant safely exemplifies reproductive justice and lactation equity in action. The shift in guidelines is a promising first step, but there is a critical need for a new paradigm that extends beyond shared decision-making. An Informed Free Choice approach seeks to promote epistemic justice, center patients' agency, and include clinicians as trusted partners who provide valuable expertise to support decision-making.

Ultimately, Informed Free Choice is not only about feeding methods; it is about restoring trust, honoring the expertise of lived experience, and ensuring that advances in HIV care translate into genuine reproductive freedom for all families affected by HIV.

## Data Availability

The original contributions presented in the study are included in the article/Supplementary Material, further inquiries can be directed to the corresponding author.
