# Correction: Navigating guidelines and realities: informed free choice in infant feeding for people living with HIV

**DOI:** 10.3389/frph.2026.1871128

**Published:** 2026-05-29

**Authors:** Laura Cox, Ciarra Covin, Monica Hahn

**Affiliations:** 1 Department of Family Health Care Nursing, School of Nursing, University of California, San Francisco, CA, United States; 2 Well Project, Philadelphia, PA, United States; 3 HIVE Clinic and Department of Family & Community Medicine, University of California, San Francisco, CA, United States

**Keywords:** breastfeeding, chestfeeding, HIV, informed free choice, reproductive justice

In the published article, an incorrect funding statement was provided. The corrected funding statement is below.

Funding

The author(s) declared that financial support was not received for this work and/or its publication. LC was supported through the T32 Next Generation Nurse Scientists Ending the HIV Epidemic Training Program (T32 NR020776) funded by the National Institute of Health. The original version of this article has been updated.

